# Impact of *The Real Cost* Campaign on Adolescents’ Recall, Attitudes, and Risk Perceptions about Tobacco Use: A National Study

**DOI:** 10.3390/ijerph14010042

**Published:** 2017-01-04

**Authors:** Li-Ling Huang, Allison J. Lazard, Jessica K. Pepper, Seth M. Noar, Leah M. Ranney, Adam O. Goldstein

**Affiliations:** 1 Lineberger Comprehensive Cancer Center, University of North Carolina, Chapel Hill, NC 27599, USA; noar@email.unc.edu (S.M.N.); adam_goldstein@med.unc.edu (A.O.G.); 2 School of Media and Journalism, University of North Carolina, Chapel Hill, NC 27599, USA; lazard@unc.edu; 3 Department of Health Behavior, Gillings School of Global Public Health, University of North Carolina, Chapel Hill, NC 27599, USA; jpepper@rti.org; 4 RTI International, Research Triangle Park, NC 27709, USA; 5 Department of Family Medicine, University of North Carolina, Chapel Hill, NC 27599, USA; leah_ranney@unc.edu

**Keywords:** smoking and tobacco use, health communication, media, outcome evaluation, health promotion

## Abstract

The Food and Drug Administration’s (FDA) *The Real Cost* campaign advertisements (ads) have targeted U.S. youth with messages designed to prevent and reduce tobacco use. This study examined exposure to *The Real Cost* campaign, including ad and slogan recall, and associations with attitudes and risk perceptions among U.S. adolescents. We analyzed data from a nationally representative sample of adolescents aged 13 to 17 years (*n* = 1125) surveyed by phone from October 2014 to June 2015. We assessed aided recall of and attitudes toward four campaign ads and the one slogan. Logistic regression models assessed whether aided recall of *The Real Cost* ads or slogan was associated with perceived likelihood of serious health consequences of cigarette smoking. Most (88%) adolescents reported seeing or hearing at least one of four ads for *The Real Cost*, and 54% recalled *The Real Cost* slogan. The majority of adolescents reported more negative attitudes toward tobacco products after seeing or hearing the ads. Recall of any *The Real Cost* ad was significantly associated with greater perceptions of serious health consequences of cigarette smoking (Adjusted Odd Ratios (AOR) = 5.58, 95% Confidence Interval (CI) = 1.20–25.90). The FDA’s *The Real Cost* campaign has achieved very high reach and is associated with more negative attitudes toward tobacco products and greater risk perceptions of cigarette smoking among U.S. adolescents.

## 1. Introduction

The 2009 Family Smoking Prevention and Tobacco Control Act gave the U.S. Food and Drug Administration (FDA) regulatory authority over tobacco products, including the responsibility of educating the public about the dangers of tobacco use. In February of 2014, FDA launched *The Real Cost*, a campaign targeting adolescents aged 12 to 17 years to prevent and reduce tobacco use by describing the true impact (or “real costs”) of tobacco use. Based on behavior change theories, *The Real Cost* campaign was developed to influence youth by increasing negative attitudes and beliefs toward tobacco use, influencing social normative beliefs, and enhancing the perceived ability to resist pressures for tobacco use [1]. Campaign messages, all including the slogan of “*The Real Cost,*” have been disseminated through television, cinema, online, radio, and print, along with social media and mobile gaming, in order to maximize reach among U.S. youth [1]. 

Understanding the effectiveness of *The Real Cost* campaign—both of the advertisements and the slogan—is key to measuring the potential impact of this investment by the FDA, as well as for developing effective future tobacco prevention mass media campaigns [2,3,4,5]. Efforts to measure campaign effectiveness can be thought of on a continuum, from ad exposure to changes in beliefs, to behavior change. Such evaluations usually begin with what proportion of the target audience has been exposed to messages (campaign reach), whether the audience remembers them (recall or recognition) [6], and whether the target audience believes them to be of high quality, appealing, or effective (perceived effectiveness) [1,7]. Without significant target audience exposure and perceptions that ads are effective, it is unlikely that a campaign will be successful. Importantly, campaign evaluations also examine whether the audience has changed their attitudes or beliefs as a results of the campaign [8]. 

Risk perceptions, such as one’s perceived probability, likelihood, or susceptibility for a negative health outcome [9], are an important predictor in theories of health behavior and thus are often included in campaign effectiveness evaluations. Higher risk perceptions may be tied to one’s willingness to engage in protective action or risky behavior [9,10,11,12]. Risk perceptions are an especially important preventive belief for addictive behavior, such as tobacco use, where initiation can easily lead to lifetime habits of tobacco use. While *The Real Cost* campaign focused on physical appearance and loss of control themes, the overriding message was that use of tobacco leads to negative outcomes, which can be captured in the context of risk perception measures.

This study used a national survey to examine adolescent recall of and responses to *The Real Cost* campaign ads and slogan (i.e., brand). We also evaluated the extent to which aided ad and slogan recall of *The Real Cost* campaign differed by individual factors, such as sociodemographic characteristics or smoking status. Finally, to determine how this campaign may be influencing U.S. adolescents, we examined whether recalled exposure to the ads was associated with risk perceptions about the serious health problems caused by cigarette smoking. 

## 2. Methods

Data were analyzed from a national sample of U.S. adolescents (*n* = 1125) surveyed on the telephone by the Carolina Survey Research Laboratory from November 2014 to June 2015. The national survey aimed to assess regulatory relevant factors and effective tobacco communication about tobacco product use, tobacco constituents, and tobacco regulatory agency credibility [13]. Two independent and non-overlapping random digit dialing frames were used to ensure coverage of approximately 98% of U.S. households [13]. The weighted sample is nationally-representative of adolescents aged 13 to 17 years living in the U.S., with cell or landline access. Interviewers obtained consent from parents or guardians and assent from adolescents on the telephone prior to conducting the survey. The four *The Real Cost* ads were broadcast before the data collection (“Tooth”: 17 March to 22 September 2014, “Skin”: 10 February to 27 October 2014, “Bully”: 10 February to 8 December 2014, “Alison”: 17 March to 22 September 2014) [1]. The Institutional Review Board at the University of North Carolina reviewed and approved study procedures (IRB # 13-2779).

## 3. Measures

### 3.1. The Real Cost Ad and Slogan Recall

For the four *The Real Cost* ads that aired in the initial phase of campaign, we assessed aided recall of the ads by giving participants a brief verbal description of an ad and then asking whether they had seen or heard of the ad (“Yes” coded as 1, “No” coded as 0). Two of *The Real Cost* ads centered on the theme of physical appearance: the “Tooth” ad where a young man pulls his tooth out with pliers and the “Skin” ad where a young woman pulls some skin off her face. Two ads centered on the theme of loss of control: the “Bully” ad where a tiny man bullies a teenager and the “Alison” ad where a high school girl sits at a lunch table and talks about a bad relationship. The campaign was launched in February 2014 (10 months prior to the survey) across multiple media platforms. Campaign ad airtime varied between 6 and 10 months, with one ad, “Bully”, on air during our data collection. We created an ad recall index (range 0–4) which indicated the number of ads that a participant recalled, which was also dichotomized to indicate whether adolescents recalled any of the four ads. We assessed aided recall of *The Real Cost* campaign slogan with the following question: “Have you ever seen or heard any ads on television or radio with the slogan *The Real Cost*?”

### 3.2. Attitudes

For participants who recalled each ad, they were asked about how they felt about tobacco products after seeing or hearing that particular ad. For participants who recalled the slogan, they were asked about how they felt about tobacco products after seeing or hearing the slogan. Responses were dichotomized into “more negative” (coded as 1) versus “more positive” or “no difference” (coded as 0) for analysis.

### 3.3. Risk Perceptions

Perceived likelihood of harm about cigarette smoking (risk perceptions) was assessed by asking all participants “If you smoked cigarettes regularly for the next 10 years, how likely do you think it is that you would develop serious health problems?” Responses were dichotomized to reflect perceived likelihood, “not at all likely” (coded as 0) vs. “somewhat likely” or “very likely” (coded as 1).

### 3.4. Smoking Status

Participants were categorized into three groups by smoking status: non-smoking adolescents who were not susceptible to using cigarettes, non-smoking adolescents who were susceptible to using cigarettes, and current cigarette users (i.e., smoked in the past 30 days). Two susceptibility questions were asked of all youth who had not used cigarettes in the past 30 days [14,15]: “Do you think you will smoke a cigarette in the next year?” and “If one of your best friends were to offer you a cigarette, would you smoke it?” Participants who chose “definitely yes”, “probably yes”, or “probably not” to any of the two questions were classified as susceptible to cigarette smoking, while participants who chose “definitely not” to both questions were classified as not susceptible. Adolescents who reported using cigarettes in the past 30 days were categorized as current cigarette users. 

### 3.5. Sociodemographics

Sociodemographic variables included sex (male, female), age (13–17 years), race (White, Black, all other races), ethnicity (Hispanic, non-Hispanic), and parental education (high school graduate or less, associate’s degree or some college, bachelor’s degree or above). 

## 4. Analysis

We analyzed survey data using SAS version 9.4 (SAS Institute Inc., Cary, NC, USA) to account for the survey design features [13]. Weighted means and percentages were generated using the PROC SURVEYMEANS and PROC SURVEYFREQ procedures to describe samples and summarize data on *The Real Cost* campaign-related measures. We used the PROC SURVEYLOGISTIC procedures for logistic regression models to examine whether sociodemographic characteristics and smoking status predicted: (a) ad recall; (b) slogan (i.e., brand) recall; and (c) negative attitudes about tobacco products in response to the ads. Finally, we used logistic regression to examine whether ad and slogan recall predicted risk perceptions about the serious health problems caused by cigarette smoking. 

## 5. Results

### 5.1. Sample Characteristics 

A national sample of adolescents (*n* = 1125) completed the survey, with a weighted response rate of 66%, which is similar to the 2014 National Youth Tobacco Survey response rate of 73% [16]. Participants were evenly distributed across ages 13–17 years (mean = 15 years old); 49% were male; 73% were White, 13% were Black, and 90% were non-Hispanic (Table 1). Smoking status showed that 83% of adolescent participants were non-susceptible nonsmokers, 14% were susceptible nonsmokers, and 3% were current cigarette smokers.

### 5.2. Recall of The Real Cost Ads and Slogan

The majority (88%) of participants reported seeing or hearing at least one of the four *The Real Cost* ads: 15% recalled just one ad, 23% recalled two ads, 28% recalled three ads, and 22% recalled all four ads. The physical appearance ad theme had higher recall rates (“Skin” 76% (95% Confidence Interval (CI): 74%–79%), “Tooth” 65% (95% CI: 62%–68%)) than the ad theme that addressed loss of control due to addiction (“Bully” 50% (95% CI: 47%–54%), “Alison” 42% (95% CI: 39%–45%)). There were no associations between ad recall and sociodemographics or smoking status (Table 2).

Over half (54%) of the participants reported recognizing the slogan *The Real Cost*. As shown in Table 2, slogan recall was significantly associated with parental education but not participant gender, age, race, ethnicity, or smoking status. Adolescents whose parents’ education was high school graduate or less were less likely to recognize the slogan than those whose parents’ education was bachelor’s degree or above (Adjusted Odds Ratio (AOR) = 0.60, 95% CI = 0.42–0.84).

### 5.3. Attitudes toward Tobacco Products

The majority of adolescents reported more negative tobacco product attitudes after seeing or hearing each of the following ads: “Skin” (79%); “Tooth” (75%); “Bully” (71%); and “Alison” (70%). Among participants who reported recall of *The Real Cost* slogan (*n* = 588), just over half (52%) reported a more negative attitude toward tobacco products after seeing or hearing the slogan. 

Having a more negative attitude toward tobacco products among adolescents who recalled *The Real Cost* slogan was associated with race and cigarette smoking status (Table 2). Black adolescents who recalled the slogan were less likely to report more negative attitudes toward tobacco products than those adolescents who were White (AOR = 0.46, 95% CI = 0.25–0.85). Among adolescents who recalled the slogan, those who were susceptible to cigarette smoking were less likely to report more negative attitudes toward tobacco products than those who were not susceptible to cigarette smoking (AOR = 0.57, 95% CI = 0.35–0.94).

### 5.4. Risk Perception about Cigarette Smoking

The majority of adolescents believed that they would develop serious health problems if they smoked cigarettes regularly for the next 10 years. In regression analysis, among adolescents who recalled at least one *The Real Cost* ad, a significantly larger percentage reported that it was somewhat or very likely that they would develop serious health problems due to regular smoking for the next 10 years compared to those who did not recall any ad (AOR = 5.58, 95% CI = 1.20–25.90) (Table 3). Slogan recall was significantly associated with risk perceptions in unadjusted models (AOR = 8.45, 95% CI = 1.02–69.99), but this association became non-significant after adjusting for sociodemographics and smoking status. 

## 6. Discussion

Tobacco use among adolescents remains a major concern in the U.S., especially given the high susceptibility of this population for initiating life-long smoking habits [17,18]. We evaluated whether the FDA’s *The Real Cost*, a national campaign to prevent and reduce smoking among adolescents aged 12 to 17 years, is reaching and potentially influencing the target audience. Evidence from this study indicates that the campaign has achieved three measures of initial success. 

First, the campaign appears to have achieved widespread reach. The large majority (88%) of adolescents recalled at least one ad from the campaign, and this was true across demographic groups. Media saturation of *The Real Cost* has effectively led to awareness well above the 75% threshold outlined as necessary to produce intended effects of a tobacco control national campaign [4,19]. This finding mirrors a recent study that showed *The Real Cost* campaign has achieved high reach and frequency of exposure among U.S. adolescents [1]. Also, high recall of the slogan “*The Real Cost*” as a single phrase of emphasis used to increase persuasiveness and cohesiveness of a campaign [20] indicated this slogan left an impression among adolescents in the U.S. This may be due to its concise and condensed nature—qualities that have been shown to increase cognitive processing of slogans or taglines [20]. In a media landscape where slogans can become cultural touchstones and outlive the campaigns of their original usage (e.g., “A Diamond is Forever”), it is important to evaluate if slogans are memorable and compelling among target audiences [20,21]. 

Second, our study shows potential campaign impact by showing how this campaign may be influencing attitudes towards tobacco use. Of those who recalled *The Real Cost* slogan or ads, over half reported more negative attitudes toward tobacco products after viewing the ads. The evidence generated from this national sample of U.S. adolescents also revealed insights for what messages are resonating with the target audience. Messages that illustrated the detrimental effects of cigarette smoking on physical health for the “Skin” and “Tooth” ads seem to have resonated more with adolescents than messages about loss of control due to addiction, based on levels of ad recall. 

Third, we found that greater *The Real Cost* ad recall was associated with greater risk perceptions about the serious health problems caused by cigarette smoking. U.S. adolescents who recalled at least one *The Real Cost* ad were five times more likely to report negative risk perceptions that they would be likely to develop serious health problems from 10 years of regular cigarette smoking. Getting adolescents to think about long-term health consequences is an important task that *The Real Cost* appears to have achieved. Non-smokers are more likely to have a better understanding of the risks to their health compared to smokers, who may minimize risks with self-exempting beliefs [11,22,23,24]. 

The strategy in *The Real Cost* messages—the use of graphic imagery to illustrate physical consequences from tobacco use—has previously been perceived as more effective than anti-industry or cessation-focused messages [7] and has been successful in the context of cigarette pack warnings [25,26]. This may be due to the higher message sensation value—the novelty of the approaches and their ability to elicit arousal [27,28]. Higher message sensation value has been shown to increase negative beliefs and impact risk behavior, especially among high sensation seekers [27,29]. While future research is needed to uncover the mechanisms that make these messages more memorable and impactful than others, our findings add to the body of literature of campaigns that discourage adolescents, and perhaps other tobacco users, from wanting to smoke [30]. These efforts are crucial as research with adolescents on effects of tobacco prevention mass media campaigns have demonstrated that ad characteristics are more important than demographics, where few differences were found [3]. Future studies should continue to evaluate message elements—source or branding of the campaign, visual design, and emotional appeals—that likely influence message reception and effectiveness [31,32,33,34]. 

This study has some limitations. While this study is the first to report on the effects of *The Real Cost* campaign on risk perceptions, the cross-sectional data collection method used here does not allow for a determination of causality. Longitudinal research that examines the impact of the campaign on perceptions of the health consequences of tobacco products, attitudes, and behavior over time will better determine directional effects. Additionally, while the phone survey allowed for a national sample to increase generalizability of the data, this limited the range of some response options for some measures and did not allow for the campaign ads to be shown to participants to assess recognition of ads. There is also a possibility that recall rates and attitudes may also have been inflated due to the social desirability of correctly answering or agreeing with the survey questions, a concern for all self-reported data. Furthermore, the small sample of participants with no recall limited the comparative analyses among those who did and did not remember the campaign. Very high percentages of adolescents had somewhat to high risk perceptions about cigarette smoking, suggesting that this risk perception measure was imperfect. Future research should measure the impact of *The Real Cost* campaign on the specific health effects targeted by the campaign. Lastly, the sample contained very few current smokers and only 14% were susceptible to smoking; thus, our findings do not provide a clear assessment of the impact of *The Real Cost* campaign on adolescent smokers.

## 7. Conclusions

We found *The Real Cost* campaign has not only achieved awareness levels deemed necessary to have a national impact, but the campaign also appears to have led to adolescents having more negative attitudes toward tobacco products after viewing the ads. Furthermore, campaign ad recall was positively associated with greater risk perceptions about adverse health problems caused by cigarette smoking among adolescents. Adolescents who remember *The Real Cost* slogan or ads think more negatively about smoking and perceive higher risks regarding the health harms associated with smoking cigarettes. These data support the messaging strategies the FDA has used in the campaign—particularly messaging that includes graphic imagery and adverse physical health effects of smoking—themes that should continue to play a central role in FDA national tobacco prevention campaigns. These strategies should also be considered for use in campaigns to educate adolescents about the risk of other tobacco products, such as e-cigarettes, newly under the jurisdiction of the FDA, as well as dual use of tobacco products [35]. While our data suggest the initial success of the campaign, further research is needed to examine the longitudinal impact of the campaign, especially its impact on smoking behavior.

## Figures and Tables

**Table 1:** Sample Characteristics (*n* = 1125).

Variable	Unweighted *n*	Unweighted % or Mean	Weighted % or Mean
Gender			
Male	561	49.9	48.7
Female	564	50.1	51.3
Age (mean)	1124	15.1	15.0
13 years	184	16.4	17.4
14 years	236	21.0	22.0
15 years	246	21.9	20.8
16 years	238	21.1	20.7
17 years	220	19.5	19.0
Race			
White	901	80.1	73.0
Black	119	10.6	13.0
All other races	105	9.3	13.9
Ethnicity			
Hispanic	84	7.4	9.8
Non-Hispanic	1040	92.6	90.2
Parental Education			
High school graduate or less	244	21.7	20.0
Associate’s degree or some college	308	27.4	27.8
Bachelor’s degree or above	571	50.9	52.2
Susceptibility to cigarette use			
Not susceptible	924	82.3	83.1
Susceptible	159	14.1	13.9
Current cigarette smoker	40	3.6	3.0

**Table 2:** Factors associated with ad recall, slogan recall, and negative attitudes toward tobacco products.

Variable	Ad Recall ^a^		Slogan Recall		Negative Attitudes toward Tobacco Products among Youth Who Recalled the Slogan	
	*n* (%)	AOR (95% CI)	*n* (%)	AOR (95% CI)	*n* (%)	AOR (95% CI)
Gender						
Male	500 (86.6)	0.68 (0.44–1.05)	322 (55.7)	1.16 (0.89–1.51)	166 (51.6)	0.92 (0.64–1.34)
Female	495 (90.5)	REF	281 (51.3)	REF	145 (52.4)	REF
Age	-	1.07 (0.92–1.26)	-	1.04 (0.95–1.15)	-	1.01 (0.89–1.15)
Race						
White	729 (88.7)	REF	450 (54.8)	REF	249 (56.1)	REF
Black	131 (89.2)	0.95 (0.51–1.76)	67 (45.5)	0.70 (0.46–1.05)	25 (37.6)	0.46 (0.25–0.85) *
All other races	136 (86.7)	0.88 (0.45–1.73)	86 (54.7)	1.11 (0.70–1.75)	26 (41.6)	0.55 (0.30–1.00)
Ethnicity						
Hispanic	94 (85.4)	0.70 (0.32–1.53)	57 (51.4)	0.92 (0.54–1.54)	26 (46.5)	0.95 (0.47–1.93)
Non-Hispanic	900 (88.8)	REF	546 (53.8)	REF	284 (52.5)	REF
Parental Education						
High school graduate or less	201 (89.4)	1.29 (0.73–2.28)	104 (46.2)	0.60 (0.42–0.84) *	56 (54.1)	1.30 (0.81–2.07)
Associate’s degree or some college	283 (90.8)	1.44 (0.86–2.40)	155 (49.7)	0.71 (0.52–0.97)	83 (54.2)	1.24 (0.79–1.92)
Bachelor’s degree or above	509 (86.9)	REF	343 (58.4)	REF	171 (50.4)	REF
Cigarette Smoking Status						
Not susceptible	821 (88.0)	REF	487 (52.2)	REF	262 (54.5)	REF
Susceptible	141 (90.1)	1.27 (0.69–2.33)	94 (59.9)	1.39 (0.95–2.03)	38 (40.5)	0.57 (0.35–0.94) *
Current cigarette smoker	33 (96.0)	2.81 (0.64–12.40)	21 (61.8)	1.62 (0.79–3.32)	10 (47.6)	0.67 (0.26–1.71)

Note. Abbreviations: AOR, adjusted odds ratio; CI, confidence interval; REF, reference group. Models adjusted for all other factors (i.e., age, sex, race, ethnicity, parental education, and cigarette smoking status). ^a^ Ad recall indicates whether adolescents recalled any of the four ads. * Statistically significant, *p* < 0.05.

**Table 3:** Association between *The Real Cost* campaign recall and risk perceptions about cigarette smoking.

Campaign-Related Measure	Risk Perceptions about Cigarette Smoking, *n* (%)	OR (95% CI)	*p*	AOR (95% CI)	*p*
Campaign ad					
No recall	126 (97.1)	REF		REF	
Recall of at least one *The Real Cost* ad	990 (99.5)	5.89 (1.36–25.45) *	0.017	5.58 (1.20–25.90) *	0.028
Campaign slogan					
No recall	515 (98.5)	REF		REF	
Recall	600 (99.8)	8.45 (1.02–69.99) *	0.047	7.18 (0.57–89.94)	0.126

Note. Abbreviations: OR, odds ratio; AOR, adjusted odds ratio; CI, confidence interval; REF, reference group. Models adjusted for sociodemographics (age, sex, race, ethnicity, parental education) and cigarette smoking status. * Statistically significant, *p* < 0.05.
